# Peer advisers compared with specialist health professionals in delivering a training programme on self-management to people with diabetes: a randomized controlled trial

**DOI:** 10.1111/j.1464-5491.2008.02542.x

**Published:** 2008-09

**Authors:** A K Baksi, M Al-Mrayat, D Hogan, E Whittingstall, P Wilson, J Wex

**Affiliations:** The Arun Baksi Centre for Diabetes and EndocrinologyLondon, UK; * Vectasearch Clinic, St Mary's Hospital, Newport, Isle of WightLondon, UK; † PharmarchitectureLondon, UK

**Keywords:** diabetes, education, peer advisers, self-management

## Abstract

**Aims:**

To assess the effectiveness and acceptability of peer advisers in diabetes in delivering a programme of training on self-management for people with diabetes.

**Methods:**

Adults with diabetes were randomly allocated to an education programme delivered either by trained peer advisers or by specialist health professionals. The primary outcome measure was change in knowledge tested before and at the conclusion of the four courses, each consisting of six sessions. Glycated haemoglobin and Diabetes Care Profile were assessed at baseline and at 6 months. Sessional and end-of-course evaluation responses were analysed, as was the attendance record.

**Results:**

Eighty-three patients were randomized. Of these, 14 failed to attend and two were excluded. Knowledge scores improved significantly in both groups, but there was no difference between the groups for any of the knowledge domains. No difference was noted in the Diabetes Care Profiles or in glycated haemoglobin. The attendance record was similar in both groups. In the post-sessional evaluations, both groups scored highly, with the health professionals significantly more so. The post-course questionnaire exploring patients’ understanding and confidence in self-management of specific aspects of diabetes care revealed no difference between the groups.

**Conclusions:**

Trained patients are as effective in imparting knowledge to their peers as specialist health professionals. Both are also acceptable to patients as trainers. However, lay tutors require to be given appropriate training, specific to the education programme they would be expected to deliver.

Diabet. Med. 25, 1076–1082 (2008)

## Introduction

National organizations recognize that a structured programme of education on self-management for people with diabetes should form the cornerstone in the management of this chronic condition [1,2]. Education has to be delivered not only at the time of diagnosis but must be continued throughout an individual's life. It is debatable whether health services have or will ever have a large enough workforce to cope with the demands of the worldwide explosion in the number of people with diabetes. Given these limitations, involving people with diabetes in the delivery of education is a logical approach to increase education provision.

Lorig *et al*. pioneered the use of lay tutors in the management of arthritis over 20 years ago [3]. Subsequently, it was recognized that other chronic diseases shared a number of similar attributes to arthritis; this gave rise to the Chronic Disease Self-Management Program (CDSMP) [4].

The formal establishment of the Expert Patient Programme (EPP) by the Department of Health confirms that the UK Government encourages the development of lay-led tutors [5]. The EPP was based on the CDSMP practised in the USA [4,6,7]. Numerous studies of the CDSMP have been reported from many countries; all of these used a mixed cohort of people with chronic disorders, including diabetes. The programmes and assessments have been generic in nature and not specific to diabetes [8–12]. The training programmes for lay tutors were either not clearly defined or were not mentioned at all [13]. Randomized studies quoted above have compared lay-led programmes with waiting-list control subjects. Two studies compared lay-led with professional-taught programmes; both were in arthritis [14,15].

In order to identify studies involving lay-led education interventions in patients with diabetes, medline, embase and Centre for Reviews and Dissemination (CRD) databases were systematically searched; no restrictions or filters were applied and no outcome measures were specified. The search identified no clinical trials specific for diabetes.

This is the first study to report a randomized controlled trial within the field of diabetes, comparing peer advisers in diabetes (PADs) with specialist health professionals (SHPs) in the delivery of patient education. PADs are people with diabetes who had undertaken special training to fulfil certain functions described elsewhere [16]. The current manuscript further describes the process of training of the PADs to be lay educators in diabetes self-management. The implications of engaging lay tutors are also discussed.

## Patients and methods

The aims of this study were to assess the effectiveness and acceptability of PADs in delivering a programme of training on self-management for people with diabetes compared with the same programme delivered by the SHPs. This was a randomized controlled trial, carried out at the Isle of Wight NHS Primary Care Trust.

A random sample of adults with diabetes aged 18 to 75 years registered at the secondary care Diabetes Centre was invited to participate in the study. Patients who agreed to participate were then randomly assigned to either the group to be taught by PADs or by the SHPs using the online QuickCalcs calculator [18]. As patients could identify instructors, blinding was not possible. Those patients who were unable to participate in a group setting (e.g. with impaired vision or hearing) and those who had already received extensive coaching (e.g. patients on insulin pump or PADs) were excluded.

Educational sessions in groups of 10 to 15 were held in the evenings, each lasting for 90 min. Four courses were conducted during the study.

The construction of the programme of training on self-management was undertaken initially with a whole day seminar of SHPs and people with diabetes and their carers. A further meeting was held with the island patient group to discuss the curriculum. This ensured that the requirements were person centred rather than being dictated by SHPs and that it was based on the needs of people with diabetes. The outcome was an educational programme with the aim of helping patients to improve their self-management. The curriculum was considered to be suitable for both diabetes Types 1 and 2. Each course would consist of six sessions held at weekly intervals. In the sixth and final session of each course, participants were to be separated according to whether they were taking insulin or were primarily on diet and/or oral agents. A lesson plan was developed for each session. This described the purpose, content and educational objectives of each session. Together with the handouts, they also served as the instructors’ manual.

The style of the teaching sessions delivered by the PADs and SHPs was the same. Sessions were interactive throughout. The format encompassed general principles and facts, along with problem solving and questions and answers. Participants were encouraged to raise issues from their own experience. Each session closed only after participants had been given the opportunity to ask questions.

At each session facilitated by PADs, a SHP was present and intervened in the event of inaccuracies. At the end of each session, participants completed an anonymized evaluation sheet; responses were on a scale of 1 to 10. They also completed an end-of-course assessment form to evaluate their confidence in specific areas of diabetes. The responses were on a scale of 1 to 5. The latter form included additional questions for patients in the PADS group to ascertain if they would have preferred to have been taught by SHP and if they felt confident with the responses given by the PADs.

### Outcome measures

The primary outcome was a change in knowledge tested before and at the conclusion of each course assessed using American Association of Clinical Endocrinologists (AACE) Knowledge Evaluation Forms [19]. This test assessed patients’ understanding of five domains—what is diabetes (18 questions), nutrition (24 questions), exercise (7 questions), monitoring (10 questions) and medications (9 questions). An attendance record was maintained. Sessional and end-of-course evaluation scores were also compared.

Secondary outcome measures were changes in glycated haemoglobin and the Diabetes Care Profile [20–22]; these were assessed at baseline and after 6 months.

### Training of PADs

PADs are people with diabetes who had voluntarily undertaken an extensive programme of training described elsewhere [16]. PADs had to complete the training and were then formally assessed by a written test followed by a 40-min oral examination conducted by independent examiners [16]. Nine PADs volunteered for further training in order to participate as lay educators in the current study. Further training of these individuals was undertaken by the SHPs, all of whom had prior training on teaching methods. The SHPs delivering the training were three specialist diabetes nurses (DH, EW and PW) and a consultant diabetologist (AKB). The nine PADs were divided into in two groups, with each group being trained by pairs of SHPs. Each training session therefore had two parallel working teams. Some PADs required more training than others.

A lesson plan was developed for each session. This described the purpose and content of the sessions. It also served as the instructors’ manual. Each session in the agreed curriculum was first delivered by a SHP and this was followed by rehearsals by the PADs. Each training session ended with all participants critiquing the performance of the presenter, with suggestions on how to improve the delivery. The presentations, handouts and lesson plans underwent several revisions during this training period. When all PADs had been assessed and felt confident with their ability to present, they underwent a dress rehearsal of an entire course before a group of invited patients. This training extended over 33 sessions.

### Statistical analysis

Calculation of the sample size required for the comparison between treatment groups was based on the only identified randomized trial of professional-led vs. lay-led education intervention in the arthritis self-management programme (ASMP) [14]. The study provided differential knowledge outcomes for lay-led and professional-led groups of arthritis patients on the scale from 0 to 10. Using 80% power and significance level α = 0.05, the minimum required sample size was determined as *n* = 31 for each arm (Stata 9.1; Stata Corp., College Sation, TX, USA). Allowance for attrition rate of 30% was made. The numbers randomized were nPAD = 40 and nSHP = 43, with 15% (randomized to PAD) and 18% (randomized to SHP) of the patients not initiating training. All patients who started the training completed the study, 34 and 33, respectively, which was more than the minimum required sample size. To also power the study for improvement in knowledge relative to baseline value regardless of the intervention, three studies identified in a recent Cochrane review of training interventions for groups of diabetic patients [23] were used. Based on these studies, using validated questionnaires and reporting knowledge scores at baseline and after 4–6 months, the minimum required sample size varied between 5 and 16 for each arm. Therefore, our study was deemed sufficiently powered for detecting within-group improvements and for comparison between interventions.

Demographic and biometric data and knowledge scores at baseline were not normally distributed and the non-parametric Mann–Whitney *U*-test was used to compare the two groups. For differences in baseline proportions, Student's *t*-test for independent samples was applied. Additionally, significance was tested for with confidence intervals around median differences using script for permutation tests (SAS 9.0; SAS Institute, Cary, NC, USA). Based on exclusion of zero, the median differences were considered significant. Non-parametric tests were used for within-group (before–after) comparisons (Wilcoxon Signed Ranked test) and for between-group comparison of before–after differences (Mann–Whitney *U*-test). Multiple linear regression was used to adjust knowledge outcomes for baseline patient characteristics; diagnostics of residuals was conducted to verify assumptions. Both forward and backward selection methods were used for robustness, with the backward selection results reported.

## Results

Of the 83 patients randomized, 40 were assigned to the PADs group and 43 to the SHPs group. Six patients in the former group and eight in the latter group failed to attend. Two patients, both in the SHP group, were excluded, one being blind and the other being on an insulin pump. Thus, 67 patients (34 in the PADs group and 33 in the SHPs group) completed the study.

There were no significant differences in baseline characteristics between patients who initiated the programme and patients who failed to attend or were excluded (*P* ≥ 0.095). Baseline demographic characteristics, type and duration of diabetes as well as the treatment regimen did not differ between the two groups; patients in the PADs group had higher body mass index (BMI) and diastolic blood pressure with a trend for higher systolic blood pressure (Table 1). In the PADs group, seven patients had retinopathy and one had neuropathy. In the SHPs group, six patients had retinopathy and three had neuropathy, rendering the difference in complication rates non-significant (*P* = 0.784). Neither was significant difference found in diabetes treatment modalities (diet, oral agents or insulin therapy) between the two groups PADs and SHPs (*P* = 0.46). Baseline knowledge of diabetes was identical in the two groups (Table 2). Course attendance was 93% in the PADs group and 95% in the SHPs group (*P* = 0.065). In the former group, 20 patients attended all six sessions, 13 attended five sessions and four patients attended four sessions, whilst in the latter group, 27 patients attended all six sessions, five attended four sessions, one attended four sessions and one attended three sessions.

**Table 1:** Baseline characteristics of patients in the two groups

Characteristic	PADs	SHPs	
Age (years)	60.5 ± 11	593 ± 13	0.865
% women	52.9	42.4	0.397
*n*/*N*	18/34	14/33	
Type 2 (%)	88.2	84.8	0.690
*n*/*N*	30/34	28/33	
% of Type 2 patients on diet	6.7	14.3	0.356
*n*/*N*	2/30	4/28	
% of Type 2 patients on oral glucose-lowering agents	66.7	67.9	0.925
*n*/*N*	20/30	19/28	
% of Type 2 patients on insulin	33.3	21.4	0.317
*n*/*N*	10/30	6/28	
Duration diabetes (years)*	12.5 (5.6–17.3)	7.6 (5.2–14.5)	0.212
BMI (kg/m^2^)	32.5 ± 5.3	28.7 ± 5.5	0.004
Systolic BP (mmHg)	141 ± 18	132 ± 17	0.068
Diastolic BP (mmHg)	79 ± 12	74 ± 10	0.043
HbA_1c_ (%)	7.6 ± 1.6	7.4 ± 1.3	0.739

Mean ± sd or

* median (25–75th centile).

BMI, body mass index; BP, blood pressure; HbA_1c_, glycated haemoglobin; *n*/*N*, actual number/total number; PADs, peer advisers in diabetes group; sd, standard deviation; SHPs, specialist health professionals group.

**Table 2:** Knowledge scores

	Peer advisers in diabetes (PADs) group					Specialist health professionals (SHPs) group					Group difference	
			
Knowledge domain	Before	After	Mean change (sd)	Median change	*P*-value (change)	Before	After	Mean change (sd)	Median change	*P*-value (change)	Unadjusted *P*-value	Adjusted *P*-value
What is diabetes	82.6	89.0	6.4 (12.1)	6.0	0.003	81.2	85.7	4.5 (16.5)	0.0	0.130	0.391	0.999
Nutrition	70.7	76.5	5.8 (10.5)	4.0	0.001	73.4	78.3	4.9 (12.8)	4.0	0.017	0.644	0.814
Exercise	74.4	88.2	13.8 (20.7)	14.0	0.001	76.2	86.6	10.4 (18.2)	14.0	0.003	0.998	0.924
Monitoring	60.7	75.2	14.5 (17.8)	10.0	< 0.001	67.6	73.1	5.5 (18.7)	0.0	0.148	0.045	0.117
Medicines	58.0	66.6	8.6 (22.9)	11.0	0.011	57.8	65.3	7.5 (15.5)	0.0	0.016	0.273	0.469
Total	71.2	78.9	7.7 (8.7)	9.0	< 0.001	72.9	78.7	5.8 (11.1)	6.0	0.002	0.777	0.797

Units are expressed as per cent.

Adjusted *P*-value: significance of group effect after adjustment for blood pressure, body mass index, age, gender, duration of diabetes and glycated haemoglobin in multiple linear regression.

sd, standard deviation.

### Knowledge results

Knowledge scores improved in all five domains in the PADs group. In the SHPs group there was improvement in three out of five domains; namely, nutrition, exercise and medicine (Table 2). Multiple linear regression showed that knowledge improvement was not significantly different in the two groups.

No significant difference was found between the PAD and SHP groups in the change of glycated haemoglobin (HbA_1c_) as a result of the interventions. The difference in HbA_1c_ change was 0.17 percentage points (*P* = 0.609). The before–after changes for each group were also non-significant with values of 0.19 (*P* = 0.429) for PADs and 0.02 (*P* = 0.915) for SHPs. Results of the Diabetes Care Profile showed no between-group difference for any of the items (Table 3).

**Table 3:** Diabetes care profile results

	Peer advisers in diabetes (PADs) group					Specialist health professionals (SHPs) group					Group difference	
			
	Before	After	Mean change (sd)	Median change	*P*-value (change)	Before	After	Mean change (sd)	Median change	*P*-value (change)	Unadjusted *P*-value	Adjusted *P*-value
Understanding	61.8	81.6	19.8 (16.7)	17.0	< 0.001	63.1	82.6	19.6 (14.6)	15.0	< 0.001	0.993	0.939
Positive attitude	65.5	68.7	3.2 (13.0)	4.0	0.178	65.7	69.5	3.8 (15.1)	4.0	0.084	0.728	0.882
Negative attitude	48.6	44.1	−4.5 (12.4)	−4.8	0.041	48.9	46.3	−2.6 (16.9)	−3.0	0.233	0.706	0.944
Self-Care ability	64.1	71.2	7.1 (14.5)	7.5	0.010	66.4	73.5	7.2 (14.6)	5.0	0.001	0.780	0.075
Importance of care	85.9	87.4	1.5 (15.4)	0.0	0.536	85.7	84.7	−1.1 (22.1)	0.0	0.649	0.730	0.388
Self-care adherence	67.9	70.3	2.3 (12.1)	2.5	0.238	73.6	77.9	4.2 (15.2)	5.0	0.023	0.415	0.281

Units are expressed as per cent.

*P*-value for adjusted group difference: significance of group effect after adjustments for blood pressure, body mass index, age, gender, duration of diabetes and glycated haemoglobin in multiple linear regression.

sd, standard deviation.

### Course evaluation

In the post-sessional evaluations both groups scored highly, with health professionals significantly more so (Table 4).

**Table 4:** Sessional evaluation scores

	PADs		SHPs		Difference		
			
Question	Mean (sd)	Median	Mean (sd)	Median	Means	Unadjusted *P*-value	Adjusted *P*-value
Q1. I feel I have learnt	8.39 (0.86)	8.25	8.94 (0.68)	9.00	0.55	0.025	0.030
Q2. Quality of presentation	8.73 (0.83)	8.85	9.22 (0.31)	9.20	0.12	0.025	0.009
Q3. Opportunities for participation	8.95 (0.51)	9.00	9.37 (0.42)	9.40	0.05	0.005	0.001
Q4. This has helped my understanding about diabetes	8.50 (0.76)	8.70	9.02 (0.49)	9.20	0.20	0.013	0.004
Q5. This will help me in managing my diabetes	8.50 (0.70)	8.60	8.89 (0.57)	9.00	0.39	0.060	0.011

PADs, peer advisers in diabetes group; sd, standard deviation; SHPs, specialist health professionals group.

Responses were on a scale of 1 (lowest) to 10 (highest).

Adjusted *P*-value: significance of group effect after adjustments for blood pressure, body mass index, age, gender, duration of diabetes and glycated haemoglobin in multiple linear regression.

The end-of-course evaluations did not show any significant differences in the perceived abilities of patients to describe aspects of diabetes and nutrition. Patients in the PADs group were asked additional questions. These included if they were happy to have been taught by peer advisers and also if they would have preferred to have been taught by SHPs. The average score for the first question was 4.5 out of 5.0, with the average score for the second question being 2.2. In addition, participants felt that the PADs had a good grasp of the subject (4.2) and they felt confident with responses given by the PADs to questions asked (4.2).

### Observations by health professionals

Health professionals had commented that the PADs did not encourage audience participation in the first course but this improved in subsequent courses. Corrections were made in the first session of course 1, when a PAD had omitted to mention insulin resistance as a cause of Type 2 diabetes. In the same course, one PAD had implied that sulphonylureas did not give rise to hypoglycaemia. Mistakes noted were not repeated by the PADs in subsequent courses.

## Discussion

The aims of the present study were to assess the effectiveness and acceptability of PADs in delivering a training programme on self-management of diabetes to fellow patients. Effectiveness was judged by direct comparison with experienced SHPs delivering the same programme to randomly assigned patients. The change in knowledge from baseline was significantly increased in both groups. However, when multiple regression analysis was used to adjust the change in knowledge for baseline variables, no difference between groups was observed. Differences in outcomes for Diabetes Care Profile and for HbA_1c_ were also not significant. The responses at the end-of-course assessments were similar in both groups, further attesting to the effectiveness of PADs in delivering training to fellow patients. In the post-sessional evaluations completed by patients, the SHPs group responses were significantly stronger, although the actual scores in the PADs group were comparable. All the SHPs were known to the patients, whereas those in the PADs group were not at all acquainted with the presenters. It is not possible to judge how these factors might have affected the scoring in the evaluation questionnaires.

All patients in both groups completed the course; there was no significant difference in the attendance record. Patients in the PADs group stated that they were happy to be taught by peer advisers. When asked if they would have preferred to have been taught by SHPs, the same group responded negatively. Patients felt that the PADs had a good grasp of the subject, were able to answer questions and had a good understanding of how it felt to be a person with diabetes. These responses indicate clear acceptance of the PADs by their peers as lay educators.

The sessions delivered by PADs were observed by SHPs. The mistakes noted by SHPs were judged not to be serious and were not repeated in subsequent courses. The presence of SHPs at sessions conducted by PADs was a requirement of the Ethics Committee. The presence of SHPs might have influenced the manner in which PADs delivered the lectures, but it is relevant to point out the SHPs kept a low profile and they intervened twice only and both interventions took place in course 1 only. Perhaps the use of some form of remote surveillance might have circumvented any effect the presence of SHPs might have had on the manner in which PADs delivered their lectures. We also undertook an analysis comparing outcomes in different courses and sessions (not reported here) and found no indication of the effect of those interventions. To make the presence of SHPs a requirement in all future courses delivered by PADs would increase the burden on health professionals and negate an important reason for trying to encourage the use of patients as a resource. PADs could teach in pairs, as in the CDSMC and EPP models. Each lay trainer could rectify the mistakes of the other as well as providing support and encouragement. PADS would be an asset to any multidisciplinary diabetes team.

The absence of any significant improvement in HbA_1c_ as a result of intervention in either group was expected and in keeping with the conclusions of a recent review of randomized controlled trials using educational interventions [24].

The findings reported above raise an important question as to what is appropriate training for patients before they are judged to be fit to undertake lay-led education for their peers. The literature on the subject of training lay tutors in diabetes is sparse. In a previous paper [16] we reported a study to train people with diabetes to be able to perform a number of different tasks, such as one-to-one consultations, and to be effective campaigners and committee members; the training programme for this was 18 weeks. This was found to be too intense. An alternative shorter training programme would be to offer a general programme in diabetes, with the object of enabling trainees to become proficient in providing one-to-one support and advice. Graduates of this programme could undertake further training should they be interested in teaching. However, it is relevant to stress that such further training would have to be tailored to the specific programme in which peer advisers would be expected to participate. In a recent review of community health workers in the USA, a wide variability in the duties and training was noted [25]. This emphasizes the need for clearer definitions of functions and training required for lay people.

We support the promotion of lay-led programmes in diabetes. Lay-led trainers should receive regular updates and appraisals, but who would or should be responsible for these remains uncertain. There are other clinical governance issues and codes of conduct to be considered; we recommend that such controls are supervised by the local group of peer advisers and supported by the local health organization.

Reluctance on the part of managers and clinicians to accept lay-led programmes has previously been discussed [12]. Healthcare systems should embark on creating an environment within society that will understand and accept the potential benefits offered by peer advisers. The UK Government's investment of £18m in the EPP [8] indicates the commitment of the state to promote lay involvement in the health service.

We conclude that trained patients are effective in delivering training programmes and they are acceptable to their peers as trainers in diabetes, provided such lay educators are given further training appropriate to the specific education programme that they would be expected to deliver. The use of peer advisers as lay educators could complement services provided by specialist health professionals. This service would be particularly relevant where resources are limited. This paper highlights the needs for further studies on the training curriculum for peer advisers in diabetes and suggests the need for clarification of issues pertaining to organizational and clinical governance.

## Abbreviations

CDSMP: Chronic Disease Self-Management Program
EPP: Expert Patient Programme
HbA_1c_: glycated haemoglobin
PADs: peer advisers in diabetes
SHPs: specialist health professionals

## References

[1] NICE (2003). Technology Appraisal Guidance 60: Guidance on the Use of Patient-Education Models for Diabetes.

[2] Department of Health (2001). National Service Framework for Diabetes.

[3] Lorig K, Lubeck D, Kraines RG, Seleznick M, Holman HR (1985). Outcomes of self-help education for patients with arthritis. Arthritis Rheum.

[4] Lorig KR, Sobel DS, Stewart AL, Brown BW, Bandura A, Ritter P (1999). Evidence suggesting that a chronic disease self-management program can improve health status while reducing hospitalization: a randomized trial. Med Care.

[5] Department of Health (2001). The Expert Patient.

[6] Bury M, Newbould J, Taylor D (2005). A Rapid Review of the Current State of Knowledge Regarding Lay-Led Self-Management of Chronic Illness.

[7] Griffiths C, Foster G, Eldridge S, Taylor S (2007). How effective are expert patient (lay-led) education programmes for chronic disease?. Br Med J.

[8] Barlow JH, Turner AP, Wright CC (2000). A randomized controlled study of the Arthritis Self-management Programme in the UK. Health Educ Res.

[9] Dongbo F, Hua F, McGowan P, Yi-e S, Lizhen Z, Huiqin Y (2003). Implementation and quantitative evaluation of chronic disease self-management programme in Shanghau, China: randomized controlled trial. Bull World Health Organ.

[10] Barlow JH, Wright CC, Turner AP, Bancroft V (2005). A 12-month follow-up study of self-management training for people with chronic disease: are changes maintained over time?. Br J Health Psychol.

[11] Griffiths C, Motlib J, Azad A, Ramsay J, Eldridge S, Feder G (2005). Randomised controlled trial of a lay-led self-management programme for Bangladeshi patients with chronic disease. Br J Gen Pract.

[12] Kennedy A, Reeves D, Bower P, Lee V, Middleton E, Richardson G (2007). The effectiveness and cost effectiveness of a national lay-led self-care support programme for patients with long-term conditions: a pragmatic randomised controlled trial. J Epidemiol Community Health.

[13] Newman S, Steed L, Mulligan K (2004). Self-management interventions for chronic illness. Lancet.

[14] Lorig K, Feigenbaum P, Regan C, Ung E, Chestain RL, Holman HR (1986). A comparison of lay-taught and professional-taught arthritis self-management courses. J Rheumtol.

[15] Cohen JL, Santer SV, de Vellis RF, deVellis BM (1986). Evaluation of arthritis self-management courses led by lay persons and by professionals. Arthritis Rheum.

[16] Baksi AK, Al-Mrayat M, Hogan D, Thomas Z, Whittingstall E, Wilson P (2005). Training programme for peer advisors in diabetes—are they the expert patients in diabetes?. Pract Diabetes Int.

[17] Department of Health, Diabetes UK (2005). Structured Patient Education in Diabetes—Report from the Patient Education Working Party.

[18] Graf Pad Software QuickCalcs online calculators for Scientists, 2002–2005. http://www.graphpad.com/quickcalcs/randomize1.cfm.

[19] American Association of Clinical Endocrinologists (2002). AACE diabetes guidelines, knowledge evaluation forms. Endocr Pract.

[20] Michigan Diabetes Research and Training Centre (2002). Survey instruments. http://www.med.umich.edu/mdrc/survey/index.html.

[21] Fitzgerald JT, Davis WK, Connell CM, Hess GE, Funnell MM, Hiss RG (1996). Development and validation of the diabetes care profile. Eval Health Prof.

[22] Anderson RM, Fitzgerald JT, Wisdom K, Davis WK, Hiss RG (1997). A comparison of global vs. disease-specific quality-of-life measures with patients having non-insulin-dependent diabetes mellitus. Diabetes Care.

[23] Deakin T, McShane CE, Cade JE, Williams RDRR (2005). Group based training for self-management strategies in people with Type 2 diabetes mellitus. Cochrane Database Syst Rev.

[24] Sigurdarottir AK, Jonsdottir H, Benediktsson (2007). Outcomes of educational interventions in Type 2 diabetes: WEKA data-mining analysis. Patient Educ Couns.

[25] Norris SL, Choudhury FM, Van hot K, Horsley T, Brownstein JN, Zhang X (2006). Effectiveness of community health workers in the care of persons with diabetes. Diabet Med.

